# Draft genome sequence of *Yersinia pseudotuberculosis* isolated from a wild rat in Japan

**DOI:** 10.1128/mra.01269-23

**Published:** 2024-04-10

**Authors:** Akiko Okutani, Masakatsu Taira, Shun Iida, Eun-Sil Park, Mikuni Tokuyoshi, Yuya Watari, Tadaki Suzuki, Ken Maeda

**Affiliations:** 1 Department of Veterinary Science, National Institute of Infectious Diseases, Tokyo, Japan; 2 Department of Pathology, National Institute of Infectious Diseases, Tokyo, Japan; 3 Graduate School of Agriculture and Life Science, The University of Tokyo, Tokyo, Japan; 4 Forestry and Forest Products Research Institute, Ibaraki, Japan; Loyola University Chicago, Chicago, Illinois, USA

**Keywords:** *Yersinia pseudotuberclosis*, wild rat, Mikura-shiima Island

## Abstract

We report a draft genome sequence of *Yersinia pseudotuberculosis* isolated from the spleen of a wild rat from Mikura-shima Island, Japan. The bacterium was identified as serotype O:4b using PCR-based O-genotyping. These genomic data provide insights into the pathogenic potential of this strain in spontaneous outbreaks among wild animals.

## ANNOUNCEMENT

*Yersinia pseudotuberculosis*, a Gram-negative enteric bacterium that causes illnesses from mild gastroenteritis to severe systemic infections in humans, is transmitted among wild animals (1). Here, spleen homogenate from a wild rat (*Rattus norvegicus*) collected in June 2022 from Mikura-shima Island, Japan (N 33°87′05″, E 139°62′98″), was inoculated intraperitoneally into two C57BL/6J mice (CLEA Japan, Inc., Tokyo, Japan). This work was approved by the Institutional Animal Care and Use Committee of NIID (No. 122180). One of the mice died on day 7 post-inoculation, presenting with enteric lesions. Genomic DNA was extracted from the spleen homogenate of the dead mouse using a QIAamp DNA Mini Kit (Qiagen, Hilden, Germany). PCR of the 16S rRNA gene used primers 341f and 806r (2) (94°C for 1 min; 28 cycles of 98°C for 10 s, 50°C for 15 s, and 68°C for 15 s). The amplicon (GenBank accession number LC801538) was sequenced on a SeqStudio Genetic Analyzer (Thermo Fisher Scientific, Waltham, MA). Taxonomic assignment as *Yersinia* sp. was made using BLAST against the NCBI 16S microbial database.

Spleen homogenate of the dead mouse was cultured on sheep blood agar. A single colony was then incubated in Luria-Bertani broth at 37°C for 24 h. Genomic DNA was extracted using the QIAamp DNA Mini Kit. PCR amplification using multiplex primers for differentiation of *Y. pseudotuberculosis* and *Y. pestis* (94°C for 30 s; 25 cycles of 94°C for 30 s, 55°C for 1 min, and 72°C for 90 s; 72°C for 10 min) amplified only 295 bp of the *inv* gene (3), indicating that the strain was *Y. pseudotuberculosis*. PCR-based O-genotyping (94°C for 2 min; 35 cycles of 94°C for 15 s, 53°C for 30 s, and 72°C for 90 s; 72°C for 5 min) (4) produced amplicons of genes *prt* (1072 bp) and *ddhAB* (407 bp). These amplicons were electrophoresed for size determination and the serotype was identified as O:4b.

A 15–18 kb library was prepared using the Single-Molecule Real-Time Cell Template Prep Kit (PacBio, Menlo Park, CA, USA) and sequenced using the PacBio Sequel II system (5). To obtain ≥99% accurate high-fidelity (HiFi) reads, adapter sequences were trimmed from the polymerase reads. A total of 57,563 HiFi reads were obtained with an N_50_ score of 10,525 bp and an average length of 9,650 bp. Default parameters were used for all software unless otherwise specified. Using Flye v2.4.2 (6), 5,210,829 bp were assembled with N_50_ value 4,497,756 bp, mean length 1,302,707 bp, and GC content 47.4 mol%. Mapping reads against the assembled contigs and polishing used Racon (original version) (7). Circularization was done using Flye v2.4.2. Putative origins of replication were identified in each circular contig with Ori-Finder 2022 (8). The final assembly yielded four contigs, consisting of one chromosome and three plasmids. The sequences were annotated using DFAST (v1.2.0) (9). Contig details are provided in Table 1.

**TABLE 1:** Genome assembly metrics and genetic features of Ypse1 contigs

Contig name	Total size (bp)	Coverage	G + C content (%)	Putative coding sequences (CDS)	tRNA genes	rRNA genes
Contig 1	4,497,756	106.6	47.5	3,882	68	22
Contig 2	502,301	99.4	47	448	13	-^*a*^
Contig 3	119,281	130.9	45.2	122	-	-
Contig 4	91,491	110.5	45.5	84	-	-

^*a*^ -, not detected.

## Data Availability

The raw reads of *Yersinia pseudotuberclosis* strain Ypse1 have been deposited in the DDBJ/GenBank with accession number DRA017201, and the genome sequences with accession numbers AP028968, AP028969, AP028970, and AP028971.
